# Associations between daily steps and cognitive function among inpatients with schizophrenia

**DOI:** 10.1186/s12888-022-03736-2

**Published:** 2022-02-04

**Authors:** Li-Jung Chen, Brendon Stubbs, I-Chia Chien, Tsuo-Hung Lan, Ming-Shun Chung, Hui-Ling Lee, Wan-Chi Hsu, Po-Wen Ku

**Affiliations:** 1 Department of Exercise Health Science, National Taiwan University of Sport, 271, Lixing Road, Taichung, 404 Taiwan; 2 Physiotherapy Department, South London and Maudsley NHS Foundation Trust, Denmark Hill, London, SE5 8AZ United Kingdom; 3 Department of Psychological Medicine, Institute of Psychiatry, Psychology and Neuroscience, King’s College London, De Crespigny Park, London, Box SE5 8AF, United Kingdom; 4 Center for the Development of Teaching and Research, Bali Psychiatric Center, Ministry of Health and Welfare, 33, Huafushan Rd, Bali Distric, New Taipei City, 249 Taiwan; 5 Tsaotun Psychiatric Center, Ministry of Health and Welfare, 161, Yu-Pin Rd, Nan-Tou County 542 Caotun Township, Taiwan; 6 Department of Medicine, National Yang Ming Chiao Tung University, Taipei, Taiwan; 7 Center for Neuropsychiatric Research, National Health Research Institutes, Miaoli, Taiwan; 8 Institute of Clinical Medicine, National Yang Ming Chiao Tung University, Taipei, Taiwan; 9 Jianan Psychiatric Center, Ministry of Health and Welfare, 80, Lane 870, Zhongshan Road, Tainan, 717 Taiwan; 10 Graduate Institute of Sports and Health Management, National Chung Hsing University, 145 Xingda Rd., South Dist, Taichung, 402 Taiwan

**Keywords:** Physical activity, Walking, Pedometer, Psychiatric disorder, Cognition

## Abstract

**Background:**

Walking is the fundamental component of taking steps and is the main form of physical activity among individuals with schizophrenia; it also offers a range of health benefits. This study aimed to examine the associations between daily steps and cognitive function and further explored how many steps were related to better cognitive function among inpatients with schizophrenia.

**Methods:**

Inpatients with schizophrenia were recruited from long-stay psychiatric wards across two hospitals (n=199 at site 1 and n=195 at site 2). Daily steps were collected with an accelerometer for 7 days. Four cognitive domains (attention, processing speed, reaction time, and motor speed) were tested at site 1, and two cognitive domains (attention and processing speed) were tested at site 2. The associations of daily steps and levels of steps/day with cognitive function were tested using multivariable linear regressions separated by site. Covariates included demographic variables, weight status, metabolic parameters, and clinical state.

**Results:**

Participants took an average of 7445 (±3442) steps/day. More steps were related to better attention, processing speed, reaction time, and motor speed after multivariable adjustments. Compared with participants taking <5000 steps/day, those taking ≥5000 steps/day showed significantly better processing speed. Participants taking ≥7500 steps/day were associated with better attention, better reaction time, and better motor speed than those taking <5000 steps/day.

**Conclusion:**

Daily steps are associated with better cognitive function among inpatients with schizophrenia. The optimal benefit for cognitive function among this clinical population is achieving 7500 steps/day or more.

**Supplementary Information:**

The online version contains supplementary material available at 10.1186/s12888-022-03736-2.

## Background

Individuals with schizophrenia tend to have higher risks of medical comorbidity and cardiovascular diseases, as well as more sedentary time and lower levels of physical activity, compared with the general population [1–3]. A sedentary lifestyle has been linked to several health problems, such as higher body mass index, lower cardiorespiratory fitness, and worse health-related quality of life, among individuals with schizophrenia [3, 4]. In contrast, a physically active lifestyle has been reported to be beneficial for this population, including better mental health and improved cognitive function and physical health [5–8]. Consequently, increasing physical activity is recognized as a key component of the wellbeing and health of people with schizophrenia [8].

To date, most physical activity research and guidelines have expressed physical activity in terms of frequency, duration, and intensity parameters. Objective devices, such as pedometers, accelerometers, and wearable technology watches, provide a new opportunity to assess and communicate physical activity in the form of daily steps [9, 10]. Most of these devices (e.g., pedometers and wearable technology watches) are simple and easy to access with relatively low cost and can easily be used in public health and clinical applications [9]. Walking, the main contributor to steps, can be performed at light, moderate, or vigorous intensity [11]. We have previously examined device-measured light and moderate-vigorous physical activity among 199 inpatients with schizophrenia [12]. The results showed positive associations between both light and moderate-vigorous physical activity and cognitive function, with a stronger relationship for light physical activity than for moderate-vigorous physical activity. Moreover, recent research has indicated that the number of steps/day is a better indicator than moderate-high intensity physical activity for cardiorespiratory fitness [13] and cognitive function [14] among patients with schizophrenia. These results suggest that recommendations to achieve a daily number of steps might be a good strategy for promoting health among patients with schizophrenia and encouraging self-management and monitoring of their activity.

Over the last two decades, step-based recommendations have emerged [9, 10, 15–19]. The common guideline of daily steps among healthy adults suggested by governments or professional organizations is achieving 10000 steps/day as the ideal goal [20–22]. Recent research has suggested that taking as few as 4400-7500 steps/day may also contribute to health benefits [18, 19, 23]. For example, taking 4400 steps/day was associated with a reduction in mortality rate, and taking 7500 steps/day had the optimal benefit for mortality reduction among older women [19]. However, these findings tend to focus on the general population. The evidence available to answer the question ‘how many steps are associated with better cognitive function among individuals with schizophrenia’ is still limited.

Therefore, we reanalyzed the previous data with regard to daily steps instead of light and moderate-vigorous physical activity [12]. We also recruited more inpatients with schizophrenia from an additional hospital, used additional tests for cognitive functions to examine the associations between daily steps and various domains of cognitive function and further explored how many steps were related to better cognitive function among inpatients with schizophrenia.

## Methods

### Participants

Participants were recruited from long-stay psychiatric wards at two mental hospitals in Taiwan (site 1: Jianan Psychiatric Center; site 2: Tsaotun Psychiatric Center). The inclusion criteria included ① age between 20 and 64 years; ② diagnosis of schizophrenia by psychiatrists relying on the criteria of the Diagnostic and Statistical Manual Disorders (*DSM-IV* at site 1, *DSM*-5 at site 2); and ③ stable and on the same antipsychotic medicine for at least three months prior to inclusion in the study. We excluded participants who were not able to communicate, walk independently, or had brain injury or neurological disorders. These conditions were collected from the hospital records and diagnosed by psychiatrists. Research assistants accompanied by psychiatric nurse practitioners (site 1) and trained occupational therapists (site 2) introduced the aim and procedure of the study at each long-stay psychiatric ward.

The sample size was calculated using G*Power by assuming an alpha level of 0.05 and a beta (power) of 0.8, with a medium effect size of .15 [24]. The results showed that a sample size of 123 would be needed to achieve a significant effect. At site 1, 200 inpatients were recruited, but one of them did not sign the informed consent form. At site 2, 218 eligible inpatients were recruited. Among them, 23 inpatients were further excluded because of refusing to take part in the study or refusing to wear the accelerometer. These resulted in 199 participants at site 1 and 195 participants at site 2 with written informed consent. Data collection was completed in 2015 at site 1 and in 2019 at site 2.

The research was approved by the Jianan Mental Hospital Institutional Review Board (site 1) and Tsaotun Psychiatric Center Institutional Review Board (site 2).

### Measures

#### Daily steps

The hospital provided 20-min (site 1) or 30-min (site 2) stretching and walking sessions in the morning and afternoon. Inpatients were also encouraged to attend a 60-min group exercise at a frequency of 5-6 times/week at site 2. These physical-activity promotion strategies, included in the usual care, were inpatients’ daily routines.

The step data were collected at both sites using a triaxial accelerometer (wActiSleep) (ActiGraph, Pensacola, FL, USA), which has been validated and utilized in prior research [2, 25]. Participants wore the accelerometer on the wrist of the nondominant hand all day for one week. They were asked to remove the accelerometer when taking a bath or performing water activities. The data were analyzed using ActiLife software version 6 (ActiGraph LLC, Pensacola, FL, USA).

#### Cognitive function

Attention (site 1). The Cognitrone test is a subset of the Vienna Test System (VTS) and has been adopted in a prior study of people with schizophrenia [26]. It measures attention and concentration by comparing four different figures about their congruence on a computer screen [27]. The total working time was 7 minutes, and the number of correct responses was calculated, with higher values representing better attention.

Reaction time and motor speed (site 1). The reaction test is also a subset of the VTS, measuring the time reacting to visual stimuli. It requires participants to put the index finger of the dominant hand on the rest key of a working panel. Then, they have to press the reaction key as quickly as possible when specific stimuli are presented. After pressing the reaction key, they should return the finger immediately to the rest key. The mean reaction time and motor speed are recorded (in milliseconds), representing the time that elapses between the start of a visual signal and the moment the finger leaves the rest key. The rest key and the reaction key were used to distinguish between reaction and motor time [26]. The higher values indicate slower reaction time.

Processing speed (site 1). The Grooved Pegboard test measures manual dexterity and processing speed and has been employed in a prior study of patients with schizophrenia [26]. The pegboard has 25 grooved holes, and the slots are randomly positioned. It requires participants to insert pegs into the board in a fixed order from side to side and from top to bottom as quickly as possible. The amount of time from when individuals start the test until the last peg is inserted in place is recorded in seconds. Each participant was tested two times with only the right hand and the left hand. Then, the average time for the two tests was computed, with higher scores suggesting slower processing speed.

Attention (site 2). Chu’s attention test assesses attention, which has shown excellent test-retest reliability (intraclass correlation coefficient (ICC) = 0.95) [28] and has been adopted in a prior study of people with schizophrenia [29]. The formal test includes 200 items and an extra 8 items for practice. There are a series of scrambled codes with the ‘*’ sign among the codes. Participants were asked to count the occurrences of the ‘*’ sign. The correct responses in 10 minutes were calculated, and a higher score represents better attention.

Processing speed (site 2). Chu's hand dexterity test assesses hand dexterity and processing speed and has been utilized in a prior study in people with schizophrenia [29]. The test-retest reliability (ICC=0.76-0.87) is acceptable [30]. The test requires participants to put an iron bar into a board in a fixed hole and to insert a nut into the iron bar. Participants needed to perform the test in a fixed order as quickly as possible within 2 minutes. Each participant was tested three times with the dominant hand, the nondominant hand, and both hands. The final score was obtained by summing the numbers of iron bars and nuts for the tests, with higher scores representing better processing speed.

#### Covariates

Demographic variables were collected, including age, sex, years of education, smoking habits, and alcohol consumption. Behaviors of smoking and alcohol consumption were classified into two levels: ‘Yes’ and ‘Never’. Body mass index (BMI) was computed from measured weight and height, and overweight/obesity was defined as BMI ≥24 [31].

Data about clinical status profiles, including illness onset, duration of hospitalization, and the use of antipsychotic medication, were retrieved from the hospital medical records. A daily equivalent dosage of chlorpromazine was computed based on records of antipsychotic medication [32].

We collected waist circumference, systolic/diastolic blood pressure (SBP/DBP), fasting glucose (FG), serum triglycerides (TG), and high-density lipoprotein cholesterol (HDL-C) from regular health checks in the hospital. Participants were classified as ‘abnormal’ if they met the following conditions: ① men with waist circumference ≥90 cm and women with waist circumference ≥80 cm; ② SBP/DBP ≥130/85 mmHg; ③ FG ≥100 mg/dl; ④ TG ≥150 mg/dl; or ⑤ men with HDL-C <40 mg/dl and women with HDL-C <50 mg/dl [33]. The index of metabolic syndromes was created by summing the number of abnormal metabolic parameters.

The tests were administered by psychiatric nurse practitioners (site 1) and trained occupational therapists (site 2) who were responsible for caring for the inpatients and had completed professional psychiatric training and research ethics training.

### Statistical analyses

Pearson correlations were used to examine the univariate associations between daily step counts and different domains of cognitive function at each site. Multivariable linear regression analyses were performed to test the independent associations between daily step counts and cognitive function. To explore how many steps were associated with better cognitive function, the number of daily steps was categorized into four levels (≥10000, 7500-9999, 5000-7499, and <5000 steps) [10, 15, 23]. Then, the variable of step levels was organized into dummy variables, where the level of steps <5000 served as the reference group. All models were adjusted for age, sex, education, smoking, alcohol consumption, weight status, illness onset, duration of hospitalization, antipsychotic medication, and number of metabolic symptoms. The level of significance was set at .05 for all statistical analyses, which were performed with SPSS statistics 22 (IBM, New York, USA).

## Results

The participants were on average aged 48 years and had been hospitalized for an average of 74.9 months with a mean illness onset of 23.9 years. The daily steps were 7,445 among inpatients with schizophrenia, with 21.6% reaching 10000 steps/day and 24.9% walking less than 5000 steps/day. Detailed information about the characteristics of the participants for each site is presented in Table 1. Differences between the two sites were noted, indicating that participants at site 2 were older and less overweight or obese and had a lower prevalence of alcohol consumption, fewer years of schooling, fewer metabolic symptoms, more hospitalization time, and more daily steps.

**Table 1:** Characteristics of participants

Variables/Mean±SD or n(%)	Site 1	Site 2	*p*
Age (year)	44.0±9.9	52.1±8.5	<.001
Sex (%)			.879
Male	122(61.3)	121 (62.1)	
Female	77 (38.7)	74 (37.9)	
Schooling (year)	11.4±2.2	10.7±2.8	.007
Smoking (%)			.136
Yes	85 (42.7)	69 (35.4)	
Never	114 (57.3)	126 (64.5)	
Alcohol (%)			.017
Yes	33 (16.6)	17 (8.7)	
Never	166 (83.4)	178 (91.3)	
BMI status (%)			<.001
Overweight/obese	81 (40.7)	43 (22.1)	
No	118 (59.3)	152 (77.9)	
Chlorpromazine equivalent doses (mg/d)	847.6±783.8	836.1±713.8	.878
Duration of hospitalization (month)	14.2±17.0	137.0±91.0	<.001
Illness onset (year)	23.8±6.5	23.9±8.1	.075
Number of Metabolic Symptoms	1.7±1.3	0.9±1.0	<.001
Daily steps (count)	6628.1±3362.2	8278.6±3329.4	<.001
Daily steps group (%)			<.001
≥10000	31 (15.6)	54 (27.7)	
7500-9999	51 (25.6)	55 (28.2)	
5000-7499	50 (25.1)	55 (28.2)	
<5000	67 (33.7)	31 (15.9)	
Cognitive performance			
Site 1: Attention ^a^ (count)	171.9±100.5		
Site 1: Processing speed ^b^ (score)	137.7±45.6		
Site 1: Reaction time ^c^ (msec)	661.8±480.3		
Site 1: Motor speed ^c^ (msec)	384.8±213.4		
Site 2: Attention ^d^ (score)		47.8±29.9	
Site 2: Processing speed ^e^ (score)		129.3±33.1	

BMI body mass index

^a^VTS-Cognitrone test, ^b^Grooved Pegboard Test, ^c^VTS-Reaction test, ^d^Chu's Attention Test, ^e^Chu's Hand Dexterity Test

Table 2 shows the univariate associations of daily steps with cognitive function among inpatients with schizophrenia separated by site. Daily steps were significantly associated with all cognitive measures at both sites, suggesting that participants with more steps/day were associated with better attention, processing speed, reaction time, and motor speed.

**Table 2:** Univariate correlations between daily steps and cognitive performance

Variables	Site 1					Site 2		
	Step counts	Attention ^a^	Processing speed ^b^	Reaction time ^c^	Motor speed ^c^	Step counts	Attention ^d^	Processing speed ^e^
Site 1								
Step counts	1							
Attention ^a^	.257^***^	1						
Processing speed ^b^	-.313^***^	-.412^***^	1					
Reaction time ^c^	-.245^***^	-.421^***^	.409^***^	1				
Motor speed ^c^	-.232^**^	-.420^***^	.236^**^	.510^***^	1			
Site 2								
Step counts						1		
Attention ^d^						.229**	1	
Processing speed ^e^						.301***	.417***	1

^a^VTS-Cognitrone test (higher values represent better attention), ^b^ Grooved Pegboard Test (higher scores suggest slower processing speed), ^c^ VTS-Reaction test (higher values indicate slower reaction time and motor speed), ^d^ Chu's Attention Test (higher scores represent better attention), ^e^ Chu's Hand Dexterity Test (higher scores represent faster processing speed)

**: *p* < 0.01, ***: *p* < 0.001 (two-tailed)

The results of multivariable linear regression analyses predicting cognitive function by counts and levels of steps/day are presented in Table 3 (Supplementary Table 1 for the results of the full model). Model 1 shows that counts of steps/day remained significantly associated with all cognitive measures even after multivariable adjustments for both sites (Site 1: R^2^ =.201, Beta=.246, *p*=.001 for attention, R^2^ =.226, Beta=-.313, *p*<.001 for processing speed, R^2^ =.120, Beta=-.267, *p*=.001 for reaction time, R^2^ =.152, Beta=-.276, *p*<.001 for motor speed; Site 2: R^2^ =.271, Beta=.259, *p*=.001 for attention, and R^2^ =.176, Beta=.232, *p*=.003 for processing speed).

**Table 3:** Results of linear regression analyses for predicting cognitive performance by counts and levels of steps/day

Variables	Site 1								Site 2			
	Attention ^a^		Processing speed ^b^		Reaction time ^c^		Motor speed ^c^		Attention ^d^		Processing speed ^e^	
	Beta	*p*	Beta	*p*	Beta	*p*	Beta	*p*	Beta	*p*	Beta	*p*
Model1												
Step counts	.246	.001	-.313	<.001	-.267	.001	-.276	<.001	.259	.001	.232	.003
R^2^	.201		.226		.120		.152		.271		.176	
Model 2 (ref: <5000 steps/day)												
≥10000	.266	.001	-.292	<.001	-.212	.015	-.247	.004	.280	.006	.330	.002
7500-9999	.230	.005	-.288	<.001	-.208	.017	-.220	.010	.212	.041	.223	.037
5000-7499	.123	.146	-.332	<.001	-.106	.241	-.165	.063	.083	.389	.265	.008
R^2^	.214		.254		.105		.145		.258		.187	

^a^ VTS-Cognitrone test (higher values represent better attention), ^b^ Grooved Pegboard Test (higher scores suggest slower processing speed), ^c^ VTS-Reaction test (higher values indicate slower reaction time and motor speed), ^d^ Chu's Attention Test (higher scores represent better attention)^e^ Chu's Hand Dexterity Test (higher scores represent faster processing speed)

Covariates: age, sex, schooling, smoking, drinking, BMI status, illness onset, hospitalization, chlorpromazine equivalents, number of metabolic symptoms

To examine how many steps were associated with better cognitive function among participants, levels of steps/day with dummy variables were also tested (Model 2). The results showed that taking ≥7500 steps/day was associated with better attention (Site 1: Beta=.230, *p*=.005 for 7500-9999 steps/day, Beta=.266, *p*=.001 for ≥10000 steps/day; Site 2: Beta=.212, *p*=.041 for 7500-9999 steps/day, and Beta=.280, *p*=.006 for ≥10000 steps/day) and better reaction time (Site 1: Beta=-.208, *p*=.017 for 7500-9999 steps/day, Beta=-.212, *p*=.015 for ≥10000 steps/day for reaction time; Beta=-.220, *p*=.010 for 7500-9999 steps/day, and Beta=-.247, *p*=.004 for ≥10000 steps/day for motor speed) in comparison to participants taking <5000 steps/day. The association between levels of steps/day and processing speed suggested that participants taking ≥5000 steps/day had better processing speed than those taking <5000 steps/day at both sites (Site 1: Beta=-.332, *p<*.001 for 5000-7499 steps/day, Beta=-.288, *p*<.001 for 7500-9999 steps/day, and Beta=-.292, *p<*.001 for ≥10000 steps/day; Site 2: Beta=.265, *p*=.008 for 5000-7499 steps/day, Beta=.223, *p*=.037 for 7500-9999 steps/day, and Beta=.330, *p*=.002 for ≥10000 steps/day).

## Discussion

To the best of our knowledge, this study is the first and largest study to explore how many steps are associated with better cognitive function among inpatients with schizophrenia. The results showed that daily steps were related to better cognitive function within each domain, including attention, reaction time, motor speed, and processing speed. Participants taking ≥5000 steps/day had better performance in one cognitive domain, namely, processing speed, compared to those with <5000 steps/day. When participants achieved 7500 steps/day or more, better cognitive function across all domains (attention, reaction time, motor speed, and processing speed) was observed.

The mean number of daily steps was 7,445 steps/day among all participants in this study, which is much higher than the general population, which has been shown to average 4961 steps/day across all age groups in 111 countries (n=717,527) [34], and higher than outpatients with schizophrenia with a mean age 26.3 years, at 5685 steps/day (n=62) [13]. One of the reasons for the high number of daily steps might be that all participants in this study were hospitalized inpatients and might be protected from daily schedules and obligations [15]. These inpatients attended 20- to 30-min walking sessions in the morning and afternoon every day at both sites. A 60-min group exercise at a frequency of 5-6 times/week was additionally provided at site 2, which may also partly explain why more daily steps were taken among participants at site 2 than at site 1.

Consistent with previous cross-sectional and longitudinal studies in older adults, daily steps were found to be associated with better cognitive function in some domains (attention, executive function, and subjective cognitive ability) [35, 36]. Among patients with schizophrenia, a 6-month follow-up study with a small sample size (n=25) found that increased walking steps were associated with greater improvements in cognitive flexibility and executive function. This study with a larger sample size also found significant associations between daily steps and better cognitive function in several domains, suggesting that walking might be beneficial for cognitive function in patients with schizophrenia, although it is a relatively mild activity in terms of intensity [14].

Regarding the question ‘How many steps/day are associated with better cognitive function among inpatients with schizophrenia?’ Researchers have suggested setting minimal values of 7000-8000 steps/day for healthy adults and 6500-8500 steps/day for individuals living with disability and/or chronic illness [9, 37]. Cavero-Redondo et al. found a proactive effect on arterial stiffness within 7,500-9,999 steps/day among adults in their review study [23]. Among middle-aged adults, a cross-sectional study examined associations of steps walked per day with brain volumes measured by magnetic resonance imaging for a large sample size (n=2354) [38]. The results showed that among individuals not meeting physical activity guidelines, achieving 7500 steps/day or more was associated with higher total brain volume. Another study found that older women taking 7500 steps/day had the optimal benefit for mortality reduction [19]. The current study showed that inpatients with schizophrenia taking ≥5000 steps/day had better performance in one cognitive domain (processing speed). However, when participants achieved 7500 steps/day or more, better performance was found in all four cognitive domains (attention, processing speed, reaction time, and motor speed). This step range fits approximately into the cutoff point of daily steps for various health benefits among the general population, with 7500 steps/day being associated with better cognitive function among inpatients with schizophrenia.

Walking is the fundamental component of taking steps, and it is the main form of physical activity among individuals with schizophrenia. It is associated with a range of health benefits, such as weight reduction and improvement of cardiorespiratory fitness and cognitive function [13, 14, 39]. Wearable devices provide quick feedback on daily steps, are simple to use, and are easy to understand. Therefore, promoting physical activity in terms of daily steps might be a feasible and useful strategy for health promotion among patients with schizophrenia [39].

Although this study involved a relatively large sample size for people with schizophrenia and measured various cognitive domains, it is subject to some limitations, which should be borne in mind when interpreting the results. The primary limitation is that the data are cross-sectional; therefore, we are not able to infer a causal relationship between daily steps and cognitive function. The absence of the control group is also a limitation. Additionally, walking intensity may affect the step-cognition association beyond the number of walking steps. All participants in this study were individuals with established psychosis who were long-term psychiatric inpatients, and the findings may not fully generalize to less severe outpatients, limiting generalizability. To extend our findings, well-designed prospective cohort studies or randomized controlled trials are required to confirm/refute our exploratory data.

In summary, daily steps are associated with better cognitive function among inpatients with schizophrenia. Taking ≥5000 steps/day shows a small but significant association with better cognitive function. The optimal benefit for cognitive function among inpatients with schizophrenia is achieving 7500 steps/day or more.

## Supplementary Information

**Additional file 1.**

## Data Availability

The datasets used and/or analyzed during the current study are available from the corresponding author on reasonable request.

## Abbreviations

BMI: Body mass index
FG: Fasting glucose
HDL-C: High-density lipoprotein cholesterol
ICC: Intraclass correlation coefficient
SBP/DBP: Systolic/diastolic blood pressure
TG: Triglycerides
VTS: Vienna Test System
