# Supplementary material for: Associations between daily steps and cognitive function among inpatients with schizophrenia
Source: BMC Psychiatry. 2022 Feb 4;22:87. doi: 10.1186/s12888-022-03736-2 (PMC8815184; doi:10.1186/s12888-022-03736-2)
Supplement: Supplementary file 1 — Additional file 1. [file 12888_2022_3736_MOESM1_ESM.docx]

**Captions for supplementary tables**

Supplementary table 1: Results of linear regression analyses for predicting cognitive performance by counts and levels of steps/day (full models)

Supplementary Table 1: Results of linear regression analyses for predicting cognitive performance by counts and levels of steps/day (full models)

| Variables | Site 1 | | | | | | | | Site 2 | | | |
| --- | --- | --- | --- | --- | --- | --- | --- | --- | --- | --- | --- | --- |
|  | Attention ^a^ | | Processing speed ^b^ | | Reaction time ^c^ | | Motor speed ^c^ | | Attention ^d^ | | Processing speed ^e^ | |
|  | Beta | *p* | Beta | *p* | Beta | *p* | Beta | *p* | Beta | *p* | Beta | *p* |
| **Model1** |  |  |  |  |  |  |  |  |  |  |  |  |
| Age | -.227 | .004 | .234 | .003 | .053 | .517 | .118 | .145 | -.047 | .566 | -.165 | .055 |
| Sex | -.016 | .844 | .056 | .488 | -.038 | .658 | -.040 | .636 | -.128 | .095 | -.067 | .401 |
| Schooling | .137 | .061 | -.123 | .088 | -.123 | .108 | -.118 | .117 | .409 | <.001 | .129 | .089 |
| Smoking ^f^ | -.028 | .748 | .098 | .249 | -.032 | .721 | -.023 | .797 | -.044 | .581 | -.046 | .579 |
| Drinking ^f^ | .076 | .322 | -.146 | .054 | -.022 | .781 | .107 | .175 | -.047 | .509 | -.094 | .209 |
| BMI ^f^ | -.179 | .030 | .030 | .712 | .146 | .093 | .045 | .593 | .021 | .799 | -.045 | .615 |
| Illness onset | .058 | .445 | -.065 | .383 | -.070 | .380 | -.154 | .050 | .103 | .196 | .178 | .032 |
| Hospitalization | -.025 | .732 | -.035 | .625 | .035 | .649 | .023 | .761 | .047 | .535 | .039 | .619 |
| Chlorpromazine | .095 | .190 | .156 | .030 | .049 | .517 | -.100 | .182 | .000 | .999 | -.156 | .035 |
| Number of MetS | .022 | .787 | .024 | .770 | .067 | .443 | -.075 | .384 | .041 | .623 | -.093 | .291 |
| Step counts | .246 | .001 | -.313 | <.001 | -.267 | .001 | -.276 | <.001 | .259 | .001 | .232 | .003 |
| R^2^ | .201 |  | .226 |  | .120 |  | .152 |  | .271 |  | .176 |  |
| **Model 2** |  |  |  |  |  |  |  |  |  |  |  |  |
| Age | -.240 | .002 | .246 | .001 | .075 | .367 | .134 | .101 | -.060 | .474 | -.181 | .036 |
| Sex | -.017 | .833 | .036 | .650 | -.047 | .594 | -.050 | .560 | -.123 | .114 | -.069 | .391 |
| Schooling | .136 | .068 | -.087 | .230 | -.119 | .132 | -.107 | .166 | .404 | <.001 | .120 | .116 |
| Smoking ^f^ | -.024 | .779 | .089 | .288 | -.029 | .752 | -.024 | .792 | -.053 | .508 | -.074 | .373 |
| Drinking ^f^ | .063 | .422 | -.166 | .030 | -.010 | .904 | .110 | .176 | -.038 | .600 | -.080 | .289 |
| BMI ^f^ | -.168 | .042 | .025 | .750 | .140 | .111 | .040 | .642 | .013 | .876 | -.058 | .519 |
| Illness onset | .061 | .428 | -.030 | .694 | -.072 | .382 | -.146 | .072 | .101 | .209 | .164 | .049 |
| Hospitalization | -.022 | .756 | -.063 | .369 | .021 | .786 | .007 | .928 | .056 | .469 | .053 | .500 |
| Chlorpromazine | .104 | .154 | .150 | .035 | .051 | .511 | -.102 | .180 | .002 | .979 | -.171 | .022 |
| Number of MetS | .040 | .639 | -.019 | .815 | .060 | .503 | -.092 | .299 | .042 | .617 | -.094 | .289 |
| Steps ^f^ |  |  |  |  |  |  |  |  |  |  |  |  |
| ≥10000 | .266 | .001 | -.292 | <.001 | -.212 | .015 | -.247 | .004 | .280 | .006 | .330 | .002 |
| 7500-9999 | .230 | .005 | -.288 | <.001 | -.208 | .017 | -.220 | .010 | .212 | .041 | .223 | .037 |
| 5000-7499 | .123 | .146 | -.332 | <.001 | -.106 | .241 | -.165 | .063 | .083 | .389 | .265 | .008 |
| R^2^ | .214 |  | .254 |  | .105 |  | .145 |  | .258 |  | .187 |  |

^a^: VTS-Cognitrone test (higher values represent better attention); ^b^: Grooved Pegboard Test (higher scores suggest slower processing speed); ^c^:VTS-Reaction test (higher values indicate slower reaction time and motor speed); ^d^: Chu's Attention Test (higher scores represent better attention); ^e^: Chu's Hand Dexterity Test (higher scores represent faster processing speed); ^f^: dummy variable

The reference of steps: <5000 steps/day
